# Antioxidant and Pro-Oxidant Capacities as Mechanisms of Photoprotection of Olive Polyphenols on UVA-Damaged Human Keratinocytes

**DOI:** 10.3390/molecules26082153

**Published:** 2021-04-08

**Authors:** Raffaella Marina Lecci, Isabella D’Antuono, Angela Cardinali, Antonella Garbetta, Vito Linsalata, Antonio F. Logrieco, Antonella Leone

**Affiliations:** 1 National Research Council, Institute of Sciences of Food Production, (CNR-ISPA, Lecce), Via Prov.le Lecce-Monteroni, 73100 Lecce, Italy; raffaellalecci@gmail.com; 2 National Research Council, Institute of Science of Food Production, (CNR-ISPA, Bari), Via Amendola, 122/O, 70126 Bari, Italy; isabella.dantuono@ispa.cnr.it (I.D.); antonella.garbetta@ispa.cnr.it (A.G.); vito.linsalata@ispa.cnr.it (V.L.); antonio.logrieco@ispa.cnr.it (A.F.L.)

**Keywords:** photoprotection, olive mill wastewater polyphenols, antioxidant activity, human keratinocytes, UVA-damage, ROS-mediated apoptosis, pro-oxidation

## Abstract

A wide variety of polyphenols are reported to have considerable antioxidant and skin photoprotective effects, although the mechanisms of action are not fully known. Environmentally friendly and inexpensive sources of natural bioactive compounds, such as olive mill wastewater (OMWW), the by-product of olive-oil processing, can be considered an economic source of bioactive polyphenols, with a range of biological activities, useful as chemotherapeutic or cosmeceutical agents. Green strategies, such as the process based on membrane technologies, allow to recover active polyphenols from this complex matrix. This study aims to evaluate the antioxidant, pro-oxidant, and photoprotective effects, including the underlying action mechanism(s), of the ultra-filtered (UF) OMWW fractions, in order to substantiate their use as natural cosmeceutical ingredient. Six chemically characterized UF-OMWW fractions, from Italian and Greek olive cultivar processing, were investigated for their antioxidant activities, measured by Trolox Equivalent Antioxidant Capacity (TEAC), LDL oxidation inhibition, and ROS-quenching ability in UVA-irradiated HEKa (Human Epidermal Keratinocytes adult) cultures. The photoprotective properties of UF-OMWW were assayed as a pro-oxidant-mediated pro-apoptotic effect on the UVA-damaged HEKa cells, which can be potentially involved in the carcinogenesis process. All the UF-OMWW fractions exerted an effective antioxidant activity in vitro and in cells when administered together with UV-radiation on HEKa. A pro-oxidative and pro-apoptotic effect on the UVA-damaged HEKa cells were observed, suggesting some protective actions of polyphenol fraction on keratinocyte cell cultures.

## 1. Introduction

Olive mill wastewater (OMWW) is a polyphenol-rich by-product of olive oil production, representing a major environmental problem in the Mediterranean countries, with an annual OMWW production that ranges between 7 and over 30 million m^3^ in the productive season [1,2,3]. For a long time, OMWW has been regarded as a hazardous waste with negative impact on the environment for its high organic load, mainly represented by the polyphenols and other organic molecules, such as lipids, sugars, fibers, and by inorganic compounds, such as metals [4,5]. Recently, this view has changed, and the OMWW are considered as a potential low-cost row material rich in bioactive compounds, particularly phenolics [6], for which concentrations range from 0.002 to 11.5 g/L [5]. They can be recovered by a green process based on the membrane technologies also reducing the organic load [7,8]. With this technology, the OMWW can be efficiently treated by using microfiltration (MF), ultrafiltration (UF), nanofiltration (NF), and/or reverse osmosis (RO), and the last permeate fraction can be discharged into aquatic systems in compliance with national and European Union (EU) regulations or be safely used for irrigation. The UF and NF permeates are microbiologically safe (as they are membrane-filtered), and dependently to the cultivar used are differently enriched in polyphenols, such as tyrosol (Tyr), hydroxytyrosol (HT), caffeic acid (CAA), *p*-coumaric acid (CUA), verbascoside (VB), isoverbascoside (isoVB), and several others minor polyphenols, with known antioxidant activity [6,7,8]. The presence of polyphenols in the UF and NF fractions define their possible application for food, pharmaceutical, and cosmetic industries, whereas MF and UF retentate have been proposed as biopesticides for their positive effect against the *Xylella fastidiosa* infection [9] and against the production of the mycotoxin Aflatoxin B1 by *Aspergillus flavus* [10]. Moreover, they can be used as fertilizers or in the production of biogas in anaerobic reactors [8].

Polyphenols represent heterogeneous classes of phytochemicals produced for plant protection against external stresses such as pathogens and UV light [11,12,13,14,15], and they are proposed as natural colorants and food preservatives in view of developing safer food additives [16]. Polyphenols play important roles in human health, acting as potent antioxidants, and have anti-inflammatory, anti-atherosclerotic, and chemo-preventive activities [17,18].

The antioxidant and anti-inflammatory activities of OMWW polyphenols are mainly attributed to the HT [19,20,21]. HT is a potent scavenger of superoxide anion and hydroxyl radical [22] and has significant antithrombotic, antiatherogenic, and anti-inflammatory activities. However, other OMWW phenolic compounds present in smaller quantities, such as VB, showed similar effects [23,24,25,26].

Several studies have shown that olive oil phenolics exert a protective in vitro effect with respect to chemically induced oxidation of human LDL, which is one of the initial steps in the onset of atherosclerosis [27]. Most studies are already performed on HT, a potent inhibitor of copper- and peroxyl radical-induced oxidation of LDL [28,29]. Other authors have assessed that the LDL peroxidation was also strongly inhibited by the addition of VB [27,30].

Phenol compounds can act as chemo-preventive agents by means of cell cycle modulation, apoptosis induction, epigenetic regulation, inhibition of signal transduction pathways such as NF-κB (nuclear factor kappa-light chain-enhancer of activated B cells), AP-1 (activator protein-1), and MAPK (mitogen-activated protein kinases), or by acting as antioxidants in countering the photocarcinogenesis [31,32,33,34].

This complex process is first induced from solar ultraviolet (UV) radiation responsible for most skin diseases, such as non-melanoma skin cancer (NMSC), which includes squamous and basal cell carcinoma (SCC and BCC), and represents the most common malignant neoplasms in humans [35]. Sunscreen characteristics have been demonstrated for many polyphenols [36] and a photo-protective action by the ability to suppress UVA-induced skin damages was exerted by many phenolic compounds on different cell types and skin [31,37,38,39,40,41,42]. Artichoke polyphenols exerted a skin anti-aging effect on endothelial cells, improving microcirculation and vasodilatation, acting as a potential anti-inflammatory and improving in vivo the roughness and elasticity [43]. For the phenolics of OMWW and their putative chemo-preventive activities, studies on HT and oleuropein showed anti-proliferative activity on MCF-7 human breast cancer cells [44]. VB showed anti-inflammatory activity on isolated murine macrophages [45] and the ability to reduce in vivo the effects of colitis induced in rats [46], including the inhibition of the enzymatic activity of matrix metalloproteinases (MMPs), which are also involved in skin aging. Tyrosol was able to restore the basal level of the anoxia-induced expression of the MMP-9 and MMP-2 and to reduce the expression of TNFα in EAhy926 human endothelial cells exposed to anoxic stress [47].

It was demonstrated that VB, isoVB, and Tyr exerted pro-oxidative activity and were able to induce ROS-mediated apoptosis in UVA-damaged human keratinocytes (HEKa) challenged from UVA rays [48]. Current observations suggest that pro-oxidant agents may represent a new class of anticancer drugs with the capacity to target tumor cells selectively. Indeed, growing evidences indicate that in both in vitro and in vivo systems, cancer cells generate excessive levels of ROS and have a state of oxidative stress, which seem to play a key role in cancer development. The role of ROS in cancer therapy is increasingly being acknowledged and the induction of oxidative stress by pro-oxidant agents, including polyphenols, is emerging as an attractive cancer-protective strategy [49,50,51,52,53,54].

In this study, UF fractions obtained by a membrane filtration system of OMWWs coming from six Italian and Greek olive cultivars were investigated for their phenolic composition, antioxidant capacity on LDL, and biologic activities on HEKa cultures. The results are useful to clarify the underlying action mechanism(s) of the effects of OMWW polyphenols and substantiate their possible use as a source of bioactive compounds functional for nutraceutical, chemotherapeutic, or cosmetic applications.

## 2. Results and Discussion

### 2.1. Total Phenol Content of UF Fractions and HPLC-DAD Analysis

**Figure 1:**
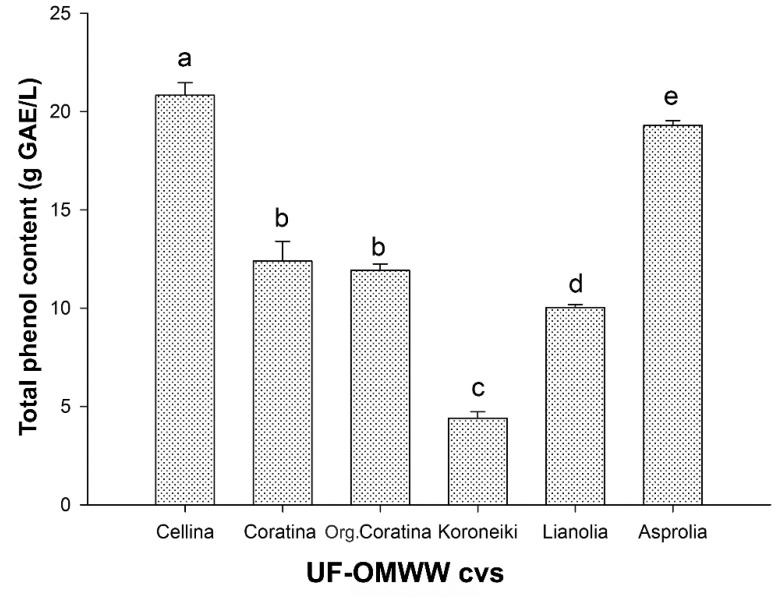
Total phenolic content (gallic acid equivalents, GAE) in UF fractions of OMWW from processing of Italian and Greek cultivar olives. Data are mean ± standard deviation (SD) of three independent experiments, each performed in triplicate. Means with different letters are significantly different.

In order to evaluate the OMWW fractions as a source of polyphenols, UF fractions coming from processed Greek and Italian olive cultivars were assayed for total phenol content by the Folin-Ciocalteu method (Figure 1). Differences in the phenolic content were found: UF fractions from *Cellina* and *Koroneiki* showed, respectively, the highest (20.83 ± 0.64 g GAE/L) and the lowest (4.41 ± 0.32 g GAE/L) phenolic content among the six UF fractions samples assayed. *Coratina* UF fractions, obtained in both traditional and organic conditions, contained 12.40 ± 1.01 g GAE/L and 11.92 ± 0.32 g GAE/L respectively, with no significant difference between the two samples. Regarding the Greek cultivars, the phenolic content in the UF fractions from *Asprolia* and *Lianolia* was 19.29 ± 0.25 g GAE/L and 10.03 ± 0.16 g GAE/L, respectively. The variability of OMWW composition is related to many parameters, such as olive variety, harvesting time, climatic conditions, and industrial process [5], however the levels of phenolic content measured in all the Italian and Greek samples seem consistent with their possible exploitation. The UF fractions utilized in this study can be considered as the best balance between the purification degree and the polyphenol enrichment, as was shown by HPLC analysis performed to characterize and quantify the main polyphenols present. As shown in Table 1, the identified phenolics were: HT, Tyr, CAA, coumaric acid (CUA), VB, IsoVB, caffeoyl-6-secologanoside (SEC), and comselogoside (COM). The most abundant compound is HT, followed by Tyr, except for UF fraction from *Coratina* olives, where VB was higher than Tyr. Conversely, the level of VB was low, and in some cases absent, in all the other samples. Interestingly, the presence of SEC and COM was detected, compounds with high antioxidant activity and already identified in both OMWW [55] and olive fruits [56]. In a recent study, carried out by the authors on the same UF fractions, twenty-three phenolic compounds were identified by LC/DAD/ESI-MSn analysis [7]. Most of them are belonging to the following classes of constituents: secoiridoids and their derivatives, phenyl alcohols, phenolic acids and their derivatives, and flavonoids [7]. Moreover, most of the identified phenols have antioxidant and other useful biological properties [27,48], and also have several scientific and economic interests, making OMWW a valuable source of natural antioxidants useful in pharmaceutical and food industries. Furthermore, the analyzed fractions were proven microbiologically safe, also due to the filtration steps, and the organic samples were free from pesticides and heavy metals contaminants (data not shown).

### 2.2. Antioxidant Activities

#### 2.2.1. Antioxidant Activity of UF Fractions

**Figure 2:**
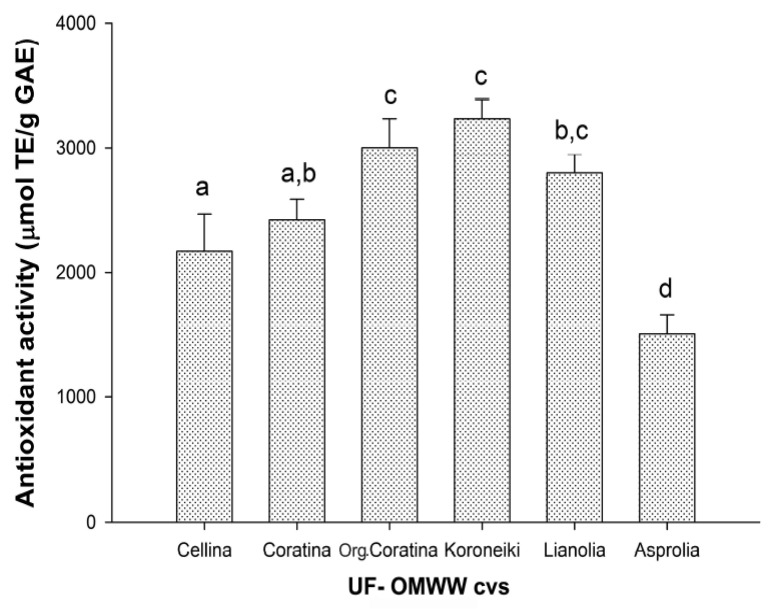
Antioxidant activity of UF fractions of OMWW from processing of Italian and Greek olive cultivars. Data are expressed as Trolox Equivalents (TE) per gram of total phenols (as GAE) and are means ± SD of three independent experiments, each performed in triplicate. Means with different letters are significantly different.

In order to verify and compare the total antioxidant activity of UF fractions, TEAC assay was used. To overcome differences linked to the different phenolic concentrations among the samples, the antioxidant activity was referred to the total phenolic content and expressed as μmol TE/g GAE, as shown in Figure 2. The highest antioxidant activities were detected in the organic *Coratina* UF fractions (3002.79 ± 230.92 μmol TE/g GAE), *Koroneiki* UF fractions (3233.23 ± 153.25 μmol TE/g GAE), and *Lianolia* UF fractions (2794.56 ± 149.04 μmol TE/g GAE), without significant differences. The lowest antioxidant activity was shown in the UF fractions from *Asprolia* (1509.87 ± 152.25 μmol TE/g GAE), followed by *Cellina* and *Coratina* UF fractions (2167.80 ± 297.62 μmol TE/g GAE and 2419.33 ± 164.41 μmol TE/g GAE, respectively). The antioxidant activity values were all similar or higher than that of single phenolic compounds evaluated with same method [57], also considering that here, they refer to the real phenolic content (GAE) rather than dry weight of the sample. When the value of total antioxidant activity was compared with the characterization of phenolic composition (Table 1), the antioxidant activity appeared to be not strictly related to specific phenolic compounds, thus this activity could be dependent from the specific heterogeneous mix of phenols acting synergistically.

#### 2.2.2. Antioxidant Activity on LDL

Table 2 shows the antioxidant effects of the six UF fractions, measured as the inhibition of hexanal formation during copper-catalyzed human LDL oxidation at three different HPLC polyphenol concentrations (Table 1).

The LDL antioxidant ability was dose-dependent in almost all samples and was different among the six cultivars. At the lower phenolic concentration (0.01 μg/mL), the percentage of inhibitions varied from 14% to 28.4% and *Lianolia* showed the highest antioxidant capacity. At 0.03 μg/mL, the six UF fractions exhibited different behavior, with the percentage of inhibitions that varied from 28.3% to 84.2%. *Koroneiki* showed the highest antioxidant activity, followed by the *Coratina,* organic *Coratina,* and *Lianolia*, while *Asprolia* and *Cellina* were less active. At the higher polyphenol concentration considered (0.1 μg/mL), all the UF fractions showed high efficiency, with an inhibition percentage greater than 60%. Among them, *Koroneiki* was again the most active, reaching 90.5% (Table 2).

**Figure 3:**
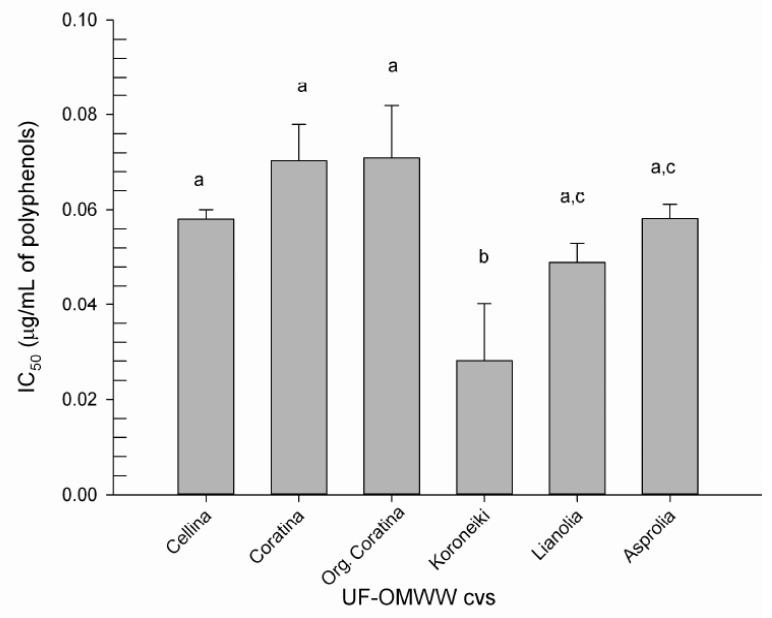
Concentration of polyphenols (μg/mL) from UF fractions of OMWW producing 50% of inhibition of LDL oxidation (IC_50_), induced by CuSO_4_. Different letters indicate difference in polyphenol concentrations between the samples (*p* < 0.05).

Figure 3 shows the IC50 values, i.e., phenolic concentration (μg/mL, Table 1) that gives 50% of inhibition in the model reaction, as interpolated by dose-response curves for the six UF fractions. As it is shown, no significant differences were found among the three Italian UF fractions and *Asprolia*, with an IC50 ranging between 0.058 ± 0.002 to 0.071 ± 0.011 μg/mL. Between the two other Greek cultivars, *Koroneiki* confirms the major potential for LDL oxidation inhibition, with an IC50 of 0.028 ± 0.012 μg/mL, followed by *Lianolia* with an IC50 of 0.049 ± 0.004 μg/mL. The results obtained in this study demonstrated the high potential for the LDL oxidation inhibition of all UF fractions from the six cultivars. To our knowledge, the only available study on the ability of OMWW to inhibit the LDL oxidation found the active concentrations at 20 ppm of total polyphenols [58], which was 200 times higher than the active concentrations found in this study. The differences could probably be related both to the preparation of OMWWs extract, and to the method utilized for the evaluation of the antioxidant capacity. Comparable studies were performed on the main polyphenols present in the OMWW, such as Tyr, CAA, HT, and VB [27,28,29,59,60,61]. It was demonstrated that the pure polyphenols exerted considerable antioxidant potency towards the copper-catalyzed human LDL oxidation in vitro, at low concentrations. However, those low concentrations were slightly higher with respect to the active concentrations found in the present work. Thus, our results support the idea that the mixture of phenolic antioxidants found in the UF fractions may interact to produce synergistic protection against LDL oxidation.

### 2.3. Possible Protective Mechanism of OMWW on Keratinocytes Cell Cultures

The putative use of OMWW as a source of chemo-preventive compounds requires to consider other useful biological activities of these products, exerted in live cells, beyond their mere antioxidant capacity. Since some polyphenols usually present in OMWW, such as Tyr, VB, and isoVB, have shown a protective effect on human keratinocytes (HEKa) cell cultures [48], here, the possible photoprotection ability of the whole UF fractions was assayed on HEKa.

#### 2.3.1. Effect of UF Fractions on HEKa Cell Viability

**Figure 4:**
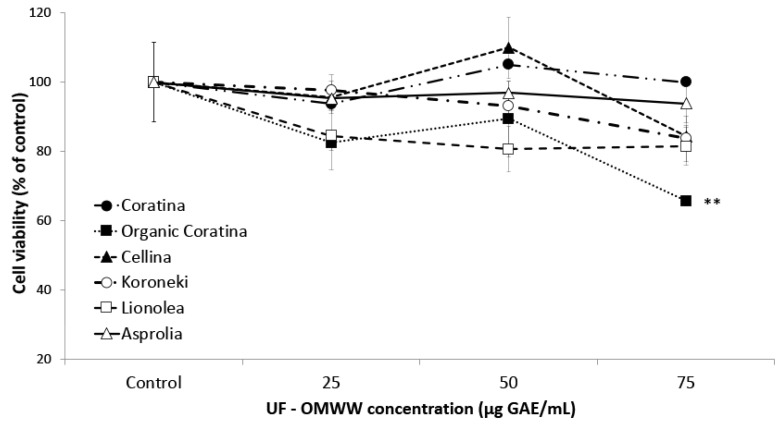
Cell viability of HEKa cultures after 16 h of treatment with UF-fractions of OMWW. Data are means ± standard deviation (SD) of three independent experiments, each performed in triplicate. ** *p* < 0.03.

In order to test the possible cytotoxic effect, all the UF fractions were assayed, at different phenol concentrations, on HEKa cultures for 16 h, during which time the substances resulted stable [48]. As showed in Figure 4, UF fractions showed no cytotoxic effect at the investigated concentration range, except for the UF-OMWW samples from organic *Coratina*, which showed a weak but significant cytotoxic effect at the highest tested concentration (75 μg GAE/mL). Therefore, the highest not toxic concentration of 50 μg GAE/mL for each UF fraction sample was used in all the following experiments.

#### 2.3.2. Effects of UF Fractions on UVA-Irradiated HEKa

**Scheme 1:**
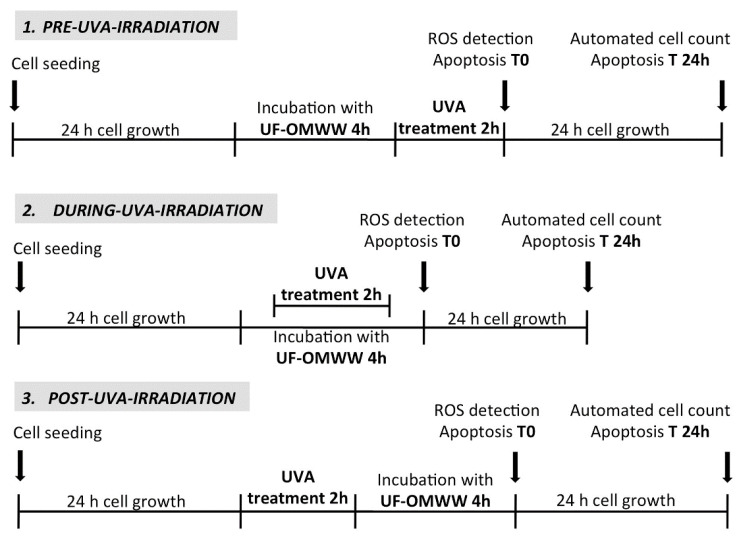
Experimental scheme of the treatments of HEKa cell cultures with Italian and Greek UF fractions and UVA irradiation (at the intensity of 20 J/cm^2^). Positive and negative controls were included in each treatment.

**Figure 5:**
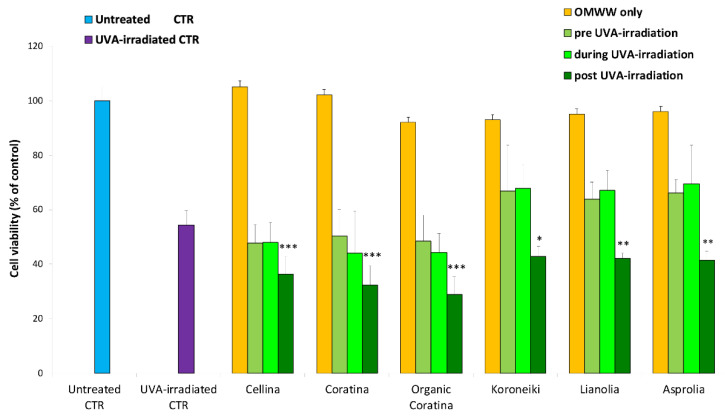
Cell viability of HEKa cultures treated as follows: HEKa cells without treatments (Untreated CTR), 2 h of UVA-irradiation followed by 24 h of growth (UVA-irradiated CTR), 4 h of treatment with UF-fraction of each of the different OMWW followed by 24 h of cell growth (OMWW only). HEKa treated with each of the different UF-OMWW before, during, or after the 2 h of UVA-irradiation (pre-UVA-irradiation, during UVA-irradiation, and post-UVA-irradiation, respectively), as outlined in Scheme 1. Data are expressed as percentage of untreated HEKa cell viability and are means ± standard deviation (SD) of three independent experiments, each performed in triplicate. * *p* < 0.05, ** *p* < 0.03, *** *p* < 0.01.

The effect of UF fractions from OMWW of Italian and Greek cultivars was investigated on HEKa irradiated with a dose of UVA rays able to affect the HEKa viability, reducing it to 60% as compared to the control (Figure 5). Three experimental treatments were performed, as described in Scheme 1, using UF fractions containing 50 μg GAE/mL of total phenols administered before (pre-UVA-irradiation), during (during-UVA-irradiation), or after (post-UVA-irradiation) the UV injury. The effect was investigated 24 h after the treatments.

##### Effect on Cell Viability of UF Fractions on UVA-Damaged HEKa

As shown in Figure 5, when the amount of 50 μg GAE/mL was added, all the Italian and Greek UF fractions exerted the same no cytotoxic effect. As compared with not treated cells, each UF fraction showed no cytotoxic effect on HEKa viability, confirming data previously obtained. The combination of OMWW and UVA treatments induced a decrease of cell viability, as compared to untreated cells (negative control), when HEKa were treated with UF-fractions before (pre-UVA-irradiation) and simultaneously (during UVA-irradiation) to the UVA-irradiation. The viability decrease ranged from 52% to 50% for Italian UF fractions and from 37% to 33% for the Greek UF fractions and showed no significant differences with the decrease of UVA-induced cell viability. When UF fractions were applied immediately after the UVA irradiation (post-UVA-irradiation), the HEKa showed a significant decrease of cell viability up to 71% for Italian samples and up to 58% for the Greek ones, as compared to the untreated controls, and up to 26% and 13% respectively, as compared to the only UVA-irradiated HEKa. The same results have been observed when HEKa cells were treated with the single pure polyphenols VB, isoVB, or Tyr, in the same experimental design [48]. Thus, these results suggested that polyphenols inside the OMWW matrices could exert the same effect of the pure polyphenols on HEKa viability. Therefore, a presumptive relation between the UF fractions effect on HEKa challenged with UVA was hypothesized, and the pro-apoptotic effect on UVA-damaged cells previously demonstrated for the pure polyphenols [48]. Thus, the possible apoptotic effect of UF fractions on HEKa treated with UVA during and after the UVA-irradiation was investigated.

##### Pro-Apoptotic Effect of UF Fractions on UVA-Damaged HEKa

**Figure 6:**
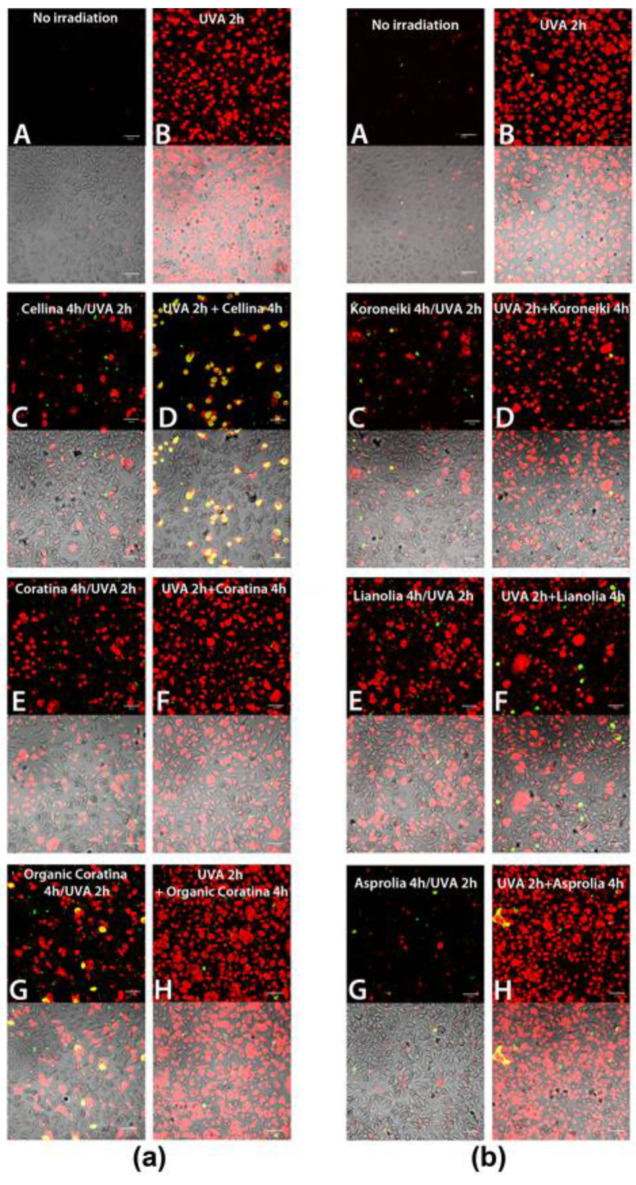
(**a**,**b**) Apoptosis detection (AnnexinV/Propidium Iodide (PI)) in HEKa cell culture subjected to treatment with UF fraction from Italian (**a**) and Greek (**b**) OMWW and UVA irradiation, soon after the treatments (T0), as outlined in Scheme 1. Red and green false colors indicate nuclei stained with PI and cells positive to Annexin V, respectively. (A) Untreated HEKa, (B) HEKa treated with only UVA, (C,E,G) HEKa treated simultaneously with UVA and UF-OMWW, (D,F,H) HEKa treated with UF-OMWW after UVA irradiation. Confocal images are representative of three independent experiments performed in triplicate, and at least 10 fields were kept as confocal images for each sample.

**Figure 7:**
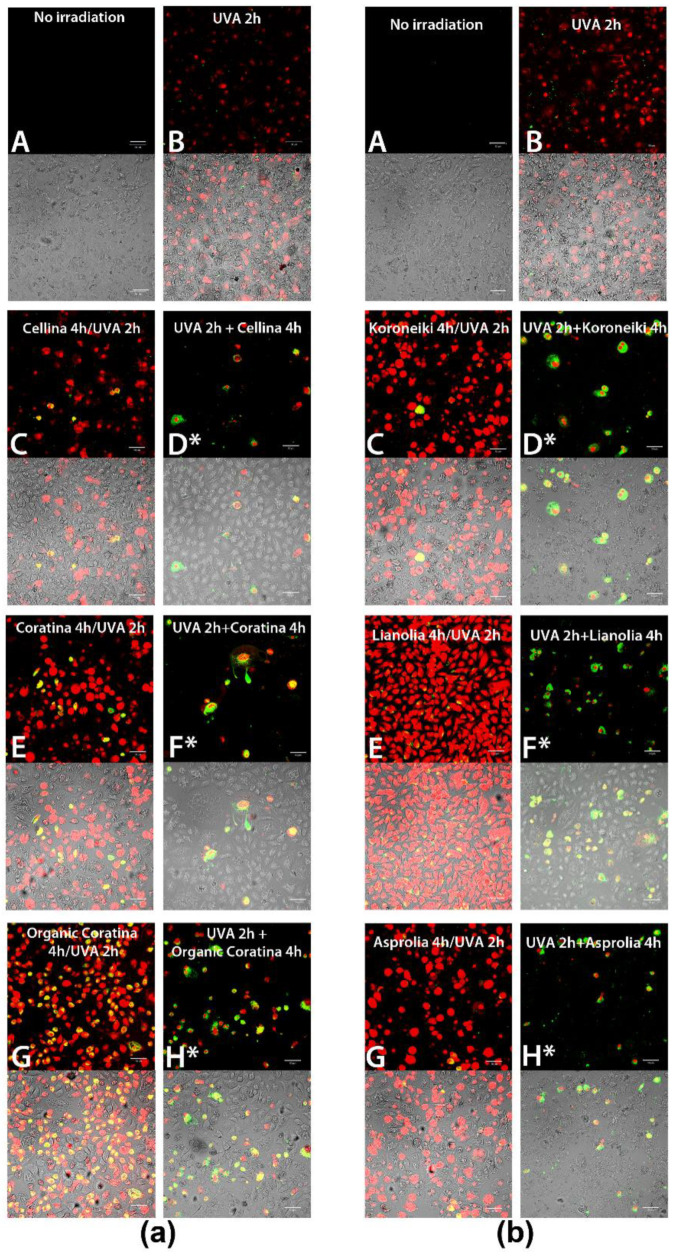
(**a**,**b**) Apoptosis detection (AnnexinV/Propidium Iodide) in HEKa cell culture subjected to treatment with UF-OMWW from Italian (**a**) and Greek (**b**) OMWWs and UVA irradiation, after 24 h of the treatments (T24), as outlined in Scheme 1. Red and green false colors indicate nuclei stained with PI and cells positive to Annexin V, respectively. (A) Untreated HEKa, (B) HEKa treated with only UVA, (C,E,G) HEKa treated simultaneously with UVA and UF-OMWW, (D,F,H) HEKa treated with UF-OMWW after UVA irradiation. (*) indicates the presence of detached and floating cells. Confocal images are representative of three independent experiments performed in triplicate, and at least 10 fields were kept as confocal images for each sample.

In order to analyze the putative pro-apoptotic effect of UF fractions on UVA-treated HEKa, Annexin-V/PI double staining assay was performed immediately after the treatments (T0) and after 24 h from the treatments (T24), and the results are shown in Figure 6 and Figure 7, respectively. No apoptotic effect was detected in not irradiated cells (Figure 6(aA,bA)) and, as expected, a dramatic necrotic effect in HEKa cells subjected to UVA irradiation was detectable soon after the treatments, as a high number of PI-stained cells (Figure 6(aB,bB)). The effect of UVA irradiation administered together with each of the six UF-OMWW on keratinocytes and detected immediately after the treatment (T0) resulted in a slightly mitigated effect of UVA injury, visible as a lower number of necrotic cells or few events of late apoptosis (detectable as PI/AnnexinV, red/green, stained cells). In particular, a certain ability to prevent the UVA-induced necrosis was observed with the *Cellina*, *Koroneiki,* and *Asprolia* samples (Figure 6(aC,bC,bG)), where *Coratina*, organic *Coratina,* and *Lianolia* showed a minor protective ability (Figure 6(aE,aG,bE)). Remarkably, the incubation with the UF-OMWW soon after UVA-irradiation was not effective; in the case of organic *Coratina* and *Asprolia,* the necrotic effect was even intensified (Figure 6(aH,bH)) and cells treated with *Cellina* (Figure 6aD) showed a number of apoptotic events. Most of the natural polyphenols are pigments able to absorb the entire UVB spectrum of wavelengths and part of the UVC- and UVA-spectra; as a consequence, when applied topically, they can prevent penetration of the radiation into the cells and, together with their antioxidant ability, can partly prevent UV-damages [62]. Conversely, when applied after the UV irradiation, compounds present in UF-OMWW make the effect on already injured cells worse, resulting in a higher number of cells undergoing necrosis, likely including only weakly or DNA-damaged cells.

A different effect was recorded when HEKa were left to grow in normal conditions for 24 h after the treatments (Figure 7a,b). UVA-irradiated keratinocytes reached a quite complete recovery and only faint necrotic events were visible (Figure 7(aB,bB)). However, a very high number of necrotic cells and late apoptotic events were still detectable in HEKa incubated with each of the six UF fractions and simultaneously subjected to UVA-injury (Figure 7(aC,aE,aG,bC,bE,bG)). Once again, HEKa treated with organic *Coratina* and *Lianolia* UF fractions during UV-challenge remained particularly affected (Figure 7(aG,bE)).

The effect of the incubation with UF fractions soon after the UVA irradiation, detected after 24 h from the treatments, was quite different: a number of apoptotic cells floating on a layer of apparently unaffected cells were detected (Figure 7(aD*,aF*,aH*, bD*,bF*,bH*)). All floating cells were apoptotic and not necrotic, and the meaning of this result seems to be related to the ability of polyphenol compounds to induce apoptosis in UV-damaged cells, thus exerting a protective action by not allowing the survival of damaged but still living cells. These results support the decrease of HEKa viability in post-UVA-irradiated samples (Figure 5) and are comparable to the strong pro-apoptotic effect demonstrated for the pure polyphenols, VB, isoVB, and Tyr, component of the OMWW, on UVA-irradiated HEKa previously observed [48]. Thus, albeit the effect of UF fractions on viability of irradiated cells was quantitatively unchanged, the cell death type was different. The effect of UVA rays and maybe the high polyphenols concentration in UF fractions, when combined, seems merely toxic when assayed after 24 h, inducing necrosis in most cells. When UVA-irradiated cells were treated with UF fractions after irradiation, a pro-apoptotic effect was detectable, presumably through triggering of specific apoptotic pathways involving Bcl-xL and Bax, as previously demonstrated for pure OMWW polyphenols [46,48]. These results can be explained if the selective pro-oxidative effect of many polyphenols is considered. Besides the literature that indicates the protective effect of several polyphenols on UV-induced apoptosis, growing evidences have explained the pro-apoptotic activity of polyphenols as a chemo-preventive and/or protective action, mainly through a ROS-dependent mechanism [48,63,64,65,66,67].

Ultraviolet radiation, together with a wide range of carcinogenic agents, induces increased level of ROS and oxidative damage in normal cells as well as in human and in animal cancers [49], including melanoma [68]. Antioxidant agents may induce cancer-preventive effects by reducing and/or preventing such increase in the cellular levels of ROS. But when pro-oxidant agents increase the cellular levels of ROS to cytotoxic levels, these agents may induce selective killing of ROS-unbalanced cells (as damaged or cancer cells) and be both preventively and therapeutically useful [51,52]. All these effects can be achieved by agents with both antioxidant and pro-oxidant properties, which can act as cancer chemo-preventive, carcinogenic, and chemotherapeutic agents, mainly depending on the concentration in which they are used. Some natural products with this double feature are drugs currently used in cancer chemotherapy or entered in clinical trials for the treatment of specific cancer, the most part are in pre-clinical models [51].

Our hypothesis is that the phenolic components of UF fractions are able to exert both antioxidant and pro-oxidant effects, as already shown for other polyphenols [50]. When administered in combination with UVA, polyphenols are weakly protective on keratinocytes, but they are able to exert pro-oxidant and pro-apoptotic effects on UVA-damaged cells when administered after the UV challenge, likely due to the higher oxidative stress in affected cells. In the latter case, they are able to reduce the survival of the damaged or putatively “initiated” cells. It is plausible that those effects may be attributable to phenolic compounds present in OMWW and concentrated in UF fractions and could be considered protective mechanisms against the survival of UVA-damaged cells potentially able to trigger carcinogenesis processes [69]. Indeed, previous results indicated that pure VB, isoVB, and Tyr, phenolic compounds usually present in OMWW, exert no toxic effects and were able to exert only the pro-apoptotic effect on UVA-irradiated HEKa [48], and HT was able to induce apoptosis in quiescent and differentiated HL60 cells [70]. Furthermore, the necrotic effect shown in HEKa soon after the treatment, versus the apoptotic effect detected 24 h after the treatments, suggested that the OMWW polyphenols act both directly on cell components and are also able to trigger apoptosis machinery for long-term reaction.

##### ROS-Quenching Ability of UF-OMWW in HEKa Cells

**Figure 8:**
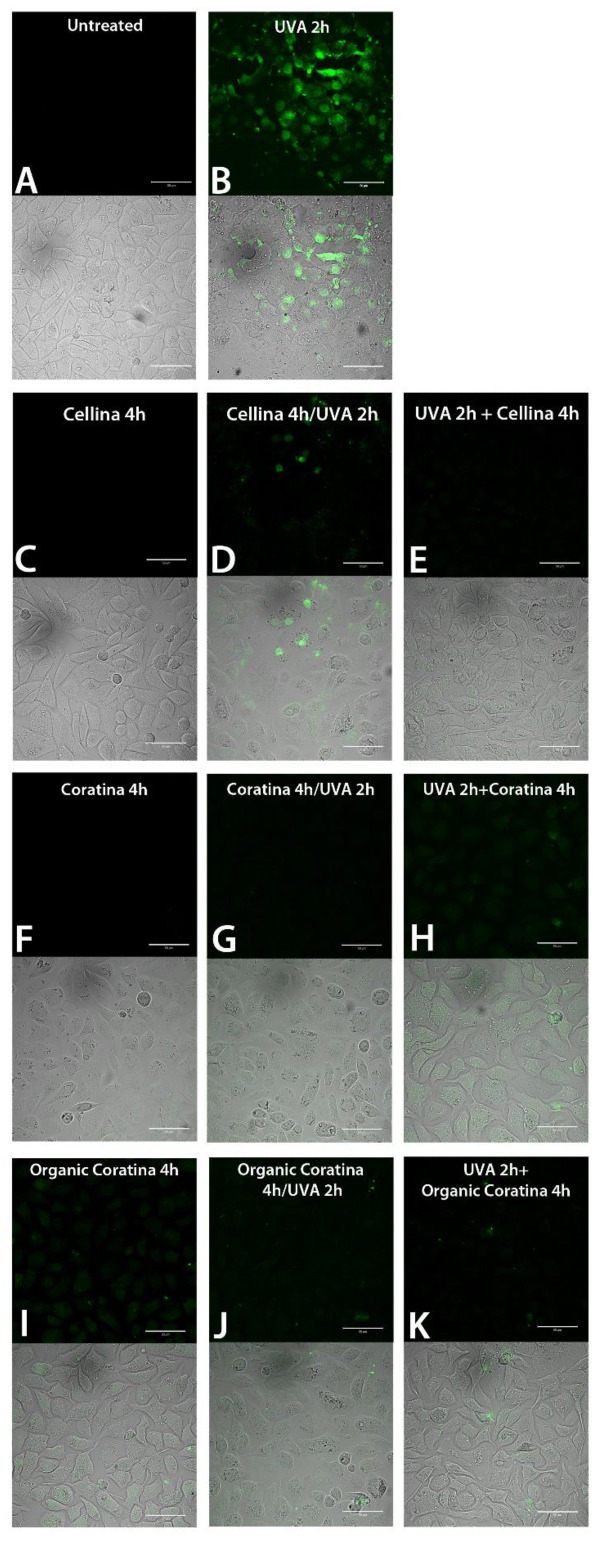
Reactive oxygen species (ROS) detection by green fluorescent probe and confocal microscopy in HEKa cell cultures subjected to treatment with UF fraction from Italian OMWWs and UVA irradiation, as outlined in Scheme 1: (**A**) Untreated HEKa, (**B**) HEKa treated with only UVA, (**C**,**F**,**I**) HEKa treated with OMWW only, (**D**,**G**,**J**) HEKa treated simultaneously with UVA and UF-OMWW, (**E**,**H**,**K**) HEKa treated with UF-OMWW after UVA irradiation. Confocal images are representative of three independent experiments performed in triplicate, and at least 12 fields were kept as confocal images for each sample.

**Figure 9:**
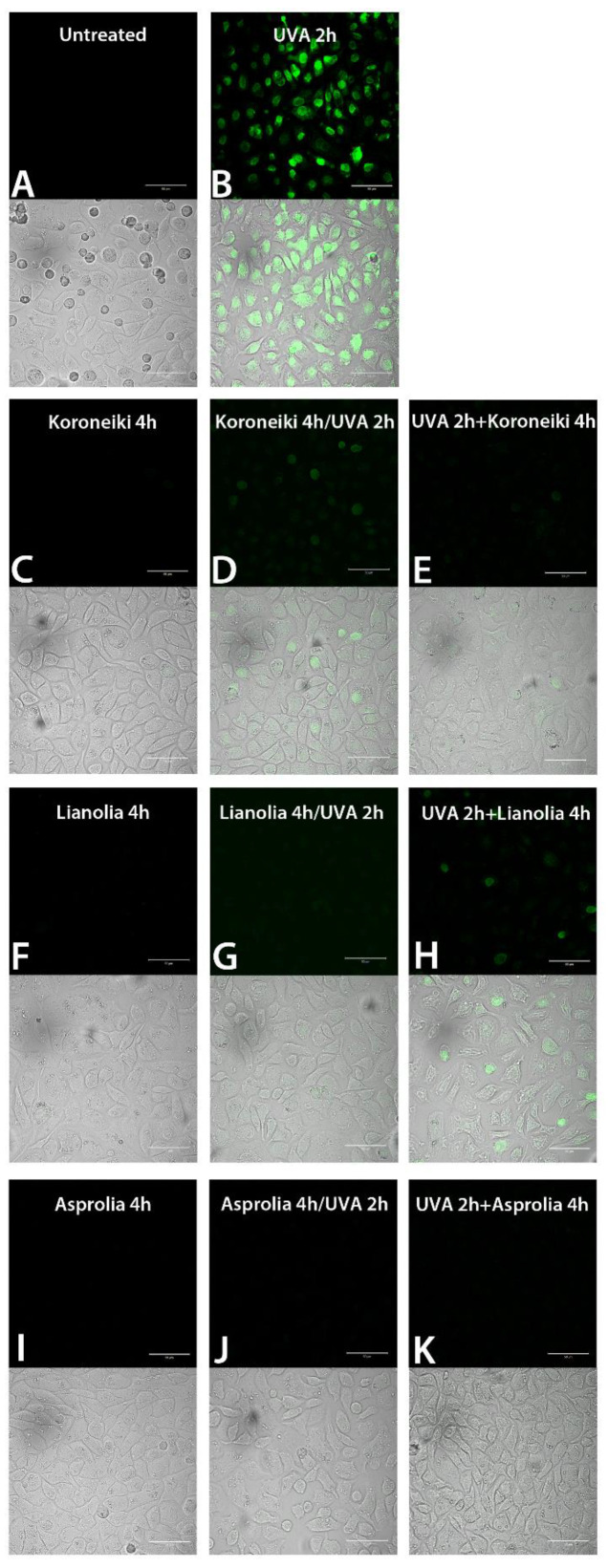
ROS detection by green fluorescent probe and confocal microscopy in HEKa cell cultures subjected to treatment with UF fraction from Greek OMWWs and UVA irradiation, as outlined in Scheme 1: (**A**) Untreated HEKa; (**B**) HEKa treated with only UVA; (**C**,**F**,**I**) HEKa treated with OMWW only; (**D**,**G**,**J**) HEKa treated simultaneously with UVA and UF-OMWW, (**E**,**H**,**K**) HEKa treated with UF-OMWW after UVA irradiation. Confocal images are representative of three independent experiments performed in triplicate, and at least 12 fields were kept as confocal images for each sample.

In order to establish if a pro-oxidative or antioxidant effect is involved in the OMWW action on HEKa, the ROS generation was qualitatively evaluated in cell cultures treated with UF fractions and UVA. No ROS generation was detected in not irradiated HEKa (Figure 8A and Figure 9A) as well as in HEKa treated only with OMWW (Figure 8C,F and Figure 9C,F,I), except for a faint effect of the organic *Coratina* OMWW (Figure 8I), while UVA induces a strong endocellular green labelling related to ROS generation (Figure 8B and Figure 9B), due to UV-induced oxidative stress [71]. All the Italian and Greek UF fractions assayed were able to attenuate the intracellular ROS formation in UVA-irradiated keratinocytes, both when administered simultaneously or after the UV treatment, suggesting an antioxidant capacity of these UF fractions also in cells. Anyway, OMWW polyphenols were not able to fully quench the oxidative stress in all the cells, as shown from few numbers of faintly fluorescent cells in almost all samples treated with UVA and UF-OMWW fractions (Figure 8 and Figure 9). UF fractions act as a mixture of potent antioxidant compounds potentially able to counteract the effect of free radicals by quenching the ROS overproduction of most of the plant-derived compounds [72,73]. Besides, some compounds can also act as pro-oxidants and then trigger a protection mechanism, eliminating damaged cells thought ROS overproduction [48,51]. To our knowledge, the ROS-quenching ability detected in UVA-irradiated HEKa, treated with UF fractions, should be attributable to the phenolic component present in those matrices. Since treatment with the single polyphenols was less efficient in the ROS quenching [48], we also have reason to believe that the complex composition in polyphenols in the OMWW matrix is a more efficient protective strategy. Taken together, the results indicated a strong antioxidant capacity of UF fractions on different in vitro systems, including LDL oxidation inhibition and cell systems, and the pro-apoptotic effect of several OMWW polyphenols on UV-damaged cells should be related to their pro-oxidant activity as a protective mechanism [53,74] against potentially initiated cells. However, the behavior of the OMWW polyphenols on live cells as well as single compounds could be more complex and requires further studies [75].

## 3. Materials and Methods

### 3.1. Chemicals

Extraction and chromatography solvents, methanol, glacial acetic acid, and ethanol, were of certified high-performance liquid chromatography (HPLC) grade, and pure standards were obtained from PhytoLab GmbH & Co. KG (Vestenbergsgreuth, Germany), Low-Density Lipoprotein (LDL) from human plasma was purchased from Sigma-Aldrich, Milan, Italy. CellROX^®^ Green Reagent and Apoptosis Kit with Annexin V Alexa Fluor^®^ 488 and Propidium Iodide were from Molecular Probes, Invitrogen (Carlsbad, CA, USA).

### 3.2. Olive Mill Wastewater (OMWW) Samples

Fresh OMWW samples were collected, among them three from Italian olive cultivars: *Cellina*, *Coratina*, and *Coratina* from organic production, obtained respectively from: Cooperativa Agricola Nuova Generazione Srl (Martano, Lecce, Italy) and Oleificio Di Molfetta (Bisceglie, Bari, Italy) from Apulia region mills, and three Greek cultivars, *Koroneiki, Lianolia,* and *Asprolia* (all organic), collected from Greek mills. Raw OMWWs were quickly subjected to treatment for recovery of phenolic substances to minimize degradation by oxidizing enzymes. OMWWs, filtered in a 500 μm test sieve, were processed with a laboratory-scale filtration system (Permeare s.r.l., Milano, Italy) consisting of a series of membranes at different porosities (0.1 and 0.05 μm) to remove the solid particles from the fluid and fractionate molecules with different molecular weight. Three types of permeated fractions were obtained: microfiltrated (MF, membrane porosity 0.1 μm), ultrafiltrated (UF, from 5000 to 200 Da), and nanofiltrated (NF, below 200 Da). The three systems use transmembrane pressures of about 2 bar for MF, 6 bar for UF, and 15 bar for NF [7]. UF fractions were utilized for the assessment of the biological activity because of their purification degree and polyphenol concentrations.

### 3.3. Determination of Phenol Content

Phenol total content was determined by the Folin-Ciocalteau colorimetric method as modified by Leo et al. [76]. After the heating at 60 °C for 1 h, 100 μL of each UF sample was mixed with 500 μL of Folin-Ciocalteau phenol reagent and with 500 μL of 7.5% sodium carbonate (Na2CO3) and left in the dark at room temperature for 2 h. The absorbance was spectrophotometrically measured at 760 nm. The calibration curve was plotted versus concentrations of gallic acid ranging from 25 to 200 μg/mL, used as a standard. The results were expressed as gallic acid equivalents (GAE) per volume of sample.

### 3.4. HPLC-DAD Analysis

Analytical-scale HPLC analyses of the UF fractions were performed with an Agilent Technologies series 1100 liquid chromatograph (Waldbronn, Germany) equipped with a binary gradient pump G1312A, a G1315A spectrophotometric photodiode array detector, and a G1316A column thermostat set at 45 °C, and Chem Station for LC3D (Rev. A. 10.02) software for spectra and data processing. An analytical Phenomenex (Torrance, CA, USA) Luna C18 (5 μm) column (4.6 × 250 mm) was used throughout this work. The solvent system consisted of (A) methanol and (B) acetic acid/water (5:95 *v*/*v*). For low molecular weight phenolics, two solvents were used: A: methanol, and B: acetic acid-water (5:95 *v*/*v*). The elution profile of the linear gradient was: 0–25 min, 15–40% A; 21–30 min, 40% A (isocratic); 30–45 min 40–63% A; 45–47 min, 63% A (isocratic), 47–51 min, 63–100% A [77]. The flow rate was 1 mL/min, and the injection volume was 20 μL.

### 3.5. Evaluation of Antioxidant Activity of UF Fractions

The ability of polyphenols presents in UF fractions to act as a natural antioxidant was examined using three different in vitro assays: two cell-free methods and one that assessed ROS (reactive oxygen species)-quenching activity in cell cultures, as described in Section 3.6.4.

#### 3.5.1. TEAC Method

The total antioxidant activity was determined spectrophotometrically by using the Trolox Equivalent Antioxidant Capacity (TEAC) method [57]. Results were expressed as μmol of Trolox equivalents per g of phenolic content (expressed as GAE) in the samples.

#### 3.5.2. LDL Antioxidant Assay

The antioxidant assay with LDL as substrate is based on measuring hexanal, one of the major end-products formed by Cu2+-catalyzed oxidation of LDL. The production of hexanal was monitored by headspace–solid phase microextration following the method of Teissedre et al. [78] with some modifications. Briefly, 50 μL of LDL samples (1 mg/mL), 320 μL of CuSO4 (80 μM), and PBS up to a final volume of 4 mL were sealed in 10 mL vials and incubated at 37 °C. At the same time, various concentrations of polyphenols from UF fractions were added to vials to determine the inhibition of copper-catalyzed LDL oxidation. The degree of inhibition of hexanal formation from LDL oxidation in the presence of antioxidant competitors was determined after 2 h of incubation at 37 °C, sampling hexanal with SPME and analyzed with the 7890 B Agilent GC system, coupled to a 240 Ion Trap Agilent spectrometer. The results, obtained after replicate analyses, were expressed as percent of relative inhibition:

% In = [(C − S)/C] × 100(1)

where C was the amount of hexanal formed in the control, and S was the amount of hexanal formed in the sample.

##### Headspace–Solid Phase Microextraction

For each OMWW cultivar (UF fractions), a polyphenols concentration ranging from 0.01 to 0.1 g/mL was achieved in the test vial. The vial was then sealed with a polypropylene cap with PTFE/silicon septum (Supelco). This mixture was stirred (280 rpm) at 37 °C for 2 h. Then, the Carboxen-PDMS (Supelco) fiber was exposed to the headspace for an additional 30 min. Afterward, the fiber was pulled into the needle sheath, and the SPME device was removed from the vial and inserted into the injection port of the gas chromatography (GC) system for thermal desorption. The same procedure was performed with a control sample containing only LDL and CuSO4.

##### Gas Chromatography–Ion Trap–Mass Spectrometry Analysis

HS-SPME analyses were performed using a 7890 B Agilent GC system, coupled to a 240 Ion Trap Agilent spectrometer and MS workstation software System Control ver. 7.0.0 (Santa Clara, CA, USA). Hexanal was separated by Agilent J&W Factor Four VF-5 ms column (30 m × 0.25 mm i.d., 0.25 μm film thickness). The injector port was heated to 250 °C. The injections were performed in split mode with a split ratio of 10:1. The carrier gas was helium 5.5 (Sapio s.r.l, Bari, Italy), at a constant flow of 1 mL min−1. The oven temperature was set at 40 °C for 2 min, then increased at 20 °C min−1 to 140 °C, and increased at 40 °C min−1 to 300 °C. All mass spectra were acquired in electron impact mode. Ionization was maintained off during the first minute. The ion trap detector was set as follows: the transfer line, manifold, and trap temperatures were 230, 50 and 150 °C, respectively. The mass ranged from *m*/*z* 25 to 150, with a scan time of 0.71 s/scan. The emission current was 30 μA, and the electron multiplier offset was set to 200 volts. The maximum ionization time was 35,000 μs. Analyses were performed in full-scan mode. Compounds were identified by comparing the retention times of the chromatographic peaks with those of authentic standards analyzed under the same conditions and by comparison of the retention indices (as Kovats index) with literature data. MS fragmentation patterns were compared with those of pure compounds, and a mass spectrum database search was performed using the National Institute of Standards and Technology (NIST V 2.0 g) spectral database. Confirmation was also conducted using a laboratory-built MS spectral database, collected from chromatographic runs of pure compounds performed with the same equipment and conditions. For quantification purposes, each sample was injected in triplicate, and the chromatographic peak areas (as k-counts amounts) of TIC were used.

### 3.6. Cell Cultures

Human Epidermal Keratinocytes adult (HEKa) cells, from Cascade Biologics^®^ (Gibco^®^, Waltham, MA, USA), were grown in Epilife Medium supplemented with 1× Human Keratinocyte Growth Supplement (HKGS), 50 U/mL of penicillin G, and 50 μg/mL of streptomycin (all from Gibco^®^, Invitrogen). Cells were plated at a density of 150,000 cell/mL in 75 cm^2^ plastic flasks, incubated at 37 °C in a 5% CO_2_ humidified atmosphere, and sub-cultured by trypsinization when 80–90% confluence was reached.

#### 3.6.1. Cell Viability Assay

HEKa cells were seeded at a density of 6 × 10^5^ cells/mL in 35 mm plates in 2 mL of the Epilife complete medium. After 24 h, when confluence was reached, HEKa cells were incubated for 16 h at 37 °C, 5% CO2 with 25, 50 and 75 μg of GAE of each UF fractions/mL. Cell viability was evaluated by Trypan blue (Invitrogen™, Carlsbad, CA, USA) dye exclusion assay associated to automated cell counting (Countess^®^ Automated Cell Counter, Invitrogen Carlsbad, CA, USA), as described in Leone et al. [79]. Three independent experiments were performed in duplicate for each treatment.

#### 3.6.2. UVA/UF-OMWW Combined Treatments

In order to test the combined effect of UF fractions treatment and UVA irradiation, HEKa cells were treated with UF fractions in PBS (to avoid sunscreen effect of medium components) [80] and UVA rays according with the protocol described in Lecci et al. [48]. Briefly, three combined treatments with UF fractions and UVA were performed, as described in Scheme 1. UF fractions were put on the cells soon before the UVA irradiation, at the same time, or soon after UVA irradiation (pre-, during, and post-UVA-irradiation, respectively). Cell viability was evaluated 24 h after the treatments. HEKa cultures treated with only medium or with only PBS and treated with only each UF fraction or only UVA-irradiated, were used as controls. Three independent experiments were performed in triplicate for each treatment.

#### 3.6.3. Annexin V/PI Staining

In order to identify apoptosis in treated and not treated HEKa cells, Annexin V-AlexaFluor488 and Propidium Iodide (PI) staining (both from Molecular Probes^®^, Invitrogen) were used following the manufacturer’s indications, as reported in Leone et al. [81]. Apoptotic cells were detected, immediately after the treatments (T0) or after 24 h of incubation at 37 °C, 5% of CO2 (T24), by a laser scanning confocal microscope (Carl Zeiss, Munich, Germany). The UVA irradiation, treatments with UF fractions, and the controls were performed according to the described protocols (Scheme 1). Viable cells were negative for both PI and Annexin V-AlexaFluor488 staining, early apoptotic cells were positive for Annexin V-AlexaFluor488 and negative for PI, and late apoptotic dead cells displayed both high Annexin V and PI labeling. Necrotic cells were positive for PI and negative for Annexin V-AlexaFluor488.

#### 3.6.4. ROS Detection in HEKa Cell Cultures

To evaluate the ROS generation, the CellROX^®^ Green Reagent (Molecular Probes^®^, Life technologies, Carlsbad, CA, USA) was used and detection was performed by a laser scanning confocal microscope (Carl Zeiss, Munchen, Germany), as described by Lecci et al. [48]. UVA irradiation, UF fraction treatments, and controls were performed as described above.

### 3.7. Statistical Analysis

Statistical analysis was performed using the analysis of variance (ANOVA) test to analyze differences between controls and each of the treatment groups. Data are mean ± standard deviation (SD). For the evaluation of the inhibition of LDL oxidation, one-way analysis of variance (ANOVA) and Student–Newman–Keuls tests were used to compare means among groups. The level of significance was set at *p* < 0.05. SigmaPlot Ver 12.0 (Sistat Software, Inc., San Jose, CA, USA) was used.

## 4. Conclusions

This study indicated that polyphenol-rich UF fractions obtained by membrane filtrated OMWW, coming from different olive cultivars and geographic zones, exerted comparable but notable biological activities: a potent antioxidant activity in different in vitro systems, including prevention of LDL oxidation and protective effects against photooxidation in human keratinocyte cultures. This last action was also exerted by a protective effect mediated by pro-oxidative ability of polyphenols, when UF-OMWW were administered after the UVA irradiation, by killing the damaged or “initiated” cells, through ROS-mediated-induction of apoptosis. Our results overall confirm the high potential for polyphenol fraction from OMWW as a source of a complex matrix of highly bioactive compounds, with advantageous features and various action mechanisms, and their possible application in nutraceutical, chemotherapeutic, or cosmetic industries.

## Figures and Tables

**Table 1:** Phenolic compositions of the UF fractions coming from Italian and Greek cultivars.

Polyphenols (µg/mL)	*Cellina*	*Coratina*	*Organic Coratina*	*Asprolia*	*Koroneiki*	*Lianolia*
Hydroxytyrosol	563.1	407.2	697.3	690.4	201.5	791.8
Tyrosol	105.6	122.0	132.0	174.3	58.0	163.8
Caffeic Acid	22.7	4.5	4.0	18.7	6.7	7.5
Cumaric Acid	3.7	2.8	3.3	37.7	6.0	4.1
Verbascoside	3.0	159.7	120.3	11.1	nd *	65.7
Isoverbascoside	nd *	19.5	15.4	nd *	nd *	nd *
Caffeoyl-6-secologanoside	nd *	5.3	nd *	18.5	4.4	4.6
Comselogoside	nd *	11.2	15.7	41.5	6.1	10.2
Total	698.1	732.2	988.0	992.2	282.7	1047.7

* nd = not detected.

**Table 2:** Inhibition of human LDL oxidation (%) by phenolic compounds from UF fractions.

Polyphenol Concentrations (µg/mL)	*Cellina*	*Coratina*	*Organic Coratina*	*Koroneiki*	*Lianolia*	*Asprolia*
0.01	20.6 ± 0.8 ^a,A^	14.0 ±1.1 ^a,A^	14.3 ± 1.2 ^a,A^	18.3 ± 2.1 ^a,A^	28.4 ± 2.7 ^a,B^	14.6 ± 0.1 ^a,A^
0.03	28.3 ± 1,0 ^b,A^	50.9 ±6.2 ^b,B^	50.4 ± 7.6 ^b,B^	84.2 ± 6.8 ^b,C^	43.7 ± 4.2 ^b,B^	29.3 ± 0.2 ^b,A^
0.1	78.3 ± 0.9 ^c,A^	64.2 ±8.1 ^b,A^	64.1 ± 9.7 ^b,A^	90.5 ± 3.2 ^b,B^	81.5 ± 7.9 ^b,B^	83.4 ± 0.6 ^c,A^

Data are given as average percent inhibition of triplicate analyses ± SD. Numbers followed by different letters (within columns in lowercase letters, within row in uppercase letters) are significantly different at *p* < 0.05.

## Data Availability

The statement can be excluded.

## Sample Availability

Samples of the Ultrafiltered Fractions of OWWWs are available from the authors.
